# Application of the DEMATEL method for quantitative analysis of risk factors for railway investments in Poland

**DOI:** 10.1371/journal.pone.0303606

**Published:** 2024-05-23

**Authors:** Jan Kowalski, Marzena Lendo-Siwicka, Zdzisław Skutnik, Dorota Mirosław-Świątek

**Affiliations:** 1 Institute of Civil Engineering, Warsaw University of Life Sciences-SGGW, Warsaw, Poland; 2 Institute of Environmental Engineering, Warsaw University of Life Sciences-SGGW, Warsaw, Poland; Chunghwa Telecom Co. Ltd., TAIWAN

## Abstract

The paper presents the results of research on the influence of risk factors on the implementation of railway investments in Poland (build stage) and deals with a detailed diagnosis of relation between factors. The application of DEcision MAking Trial and Evaluation Laboratory (DEMATEL) method for the analyses allowed to develop a cause-and-effect model of key factors and analyse the importance of the factors. Eleven factors identified in eariel studies as the most important risk factors were examined. It was found that the factors: errors in the preparation of tender documents (10.38%), errors in project documentation (10.02%), improperly estimated time of completion of the investment by the Employer (9.82%), internal regulations of PKP Polskie Koleje Państwowe S.A. (Polish State Railways) not coordinated with the provisions of contracts (9.51%) have the highest degree of importance. Factors: too many external institutions involved in the investment process and internal regulations of PKP Polskie Koleje Państwowe S.A. (Polish State Railways) not coordinated with the provisions of contracts, have the greatest net impact on the other factors. The relations between the factors and factors importance are valuable knowledge for engineers, enabling the project to be implemented according to the planned schedule and investment cost.

## Introduction

Soon, funds from the European Union intended for improving the infrastructure of member countries will no longer be directed to Poland. They will be sent to other countries, e.g. Ukraine and the Balkan countries. Therefore, if we want to effectively use the financial resources currently allocated to Poland, we must be able to predict the causes of threats that may occur during the implementation of railway investments and prepare appropriately to counteract emerging risk factors. Experience from the first tranches of financing for Polish investments shows that during the implementation of railway investments, domestic and foreign contractors encountered several problems that had not been taken into account before. Therefore, all possible remedial measures should be taken immediately to ensure that the allocated funds are spent in accordance with the adopted plan. The knowledge gathered in this area can be used when implementing similar investments in other countries.

Most rail projects are major engineering projects with complex construction technology, variable construction environments, which generate various hazard during construction process. There are a lot of example in the literature where the authors analyse different types of risk associated with railway projects and present varius methods for assessing them. In general, risk is estimated as the outcome of the probability of an adverse event and a measure of the consequences of such an event. The International Organisation for Standardisation (ISO) (2018) defined risk as the impact of uncertainty on objectives. In terms of a construction project, risk is described as any action or event that will impact the achievement of project objectives [1].

Wang et al. [2] developed a new safety risk identification method based on the on the grid–time–work breakdown structure–risk breakdown structure (G–T–WBS–RBS) matrix by integrating the methods of WBS, RBS, and risk management grid. This study proposed to move away from identifying risk factors from a static perspective to considering the dynamic process of risk factor change. Based on the spatial and temporal distribution and interaction of risk factors, the causes of risks can be examined to form more oriented risk control measures according to the characteristics of safety risks. Kowalski and Polonski [3] developed a risk estimation method (the Railway Matrix of Risk Factors (RMRF)) on Polish railway investments, based on a risk matrix which is developed at the design and construction works. Risk factors were analysed in the context of delays in investment realisation and investment costs. Risk matrix elements are represented by the calculated weight of individual risk factors. Leśniak and Janowiec used Bayesian Belief Network (BBN) for risk assessment of additional works in railway construction investment [4]. The nodes of the network are the identified risk factors. The use of BBN allows not only to estimate the risks, but also to manage them. Using the network makes it possible to compare different ways of reducing risks, to check the effect of risk factor reduction and to determine a satisfactory level of financial and time effects as a result of additional work.

Regardless of the adopted risk estimation method, a key element of the analysis is the identification of risk factors [5]. Issues related to the identification of risk factors occurring in construction are discussed broadly in literature [6]. There are several types of risk in a construction project, which can be categorised into the following types: environmental, financial, logistical, managerial, socio-political and technical [7]. In the literature there is a number of studies that identified risk factors associated with safety, delays in construction completion dates and increases in investment costs [8,9]. The most common group of factors irrespective of the country are problems related to contract management [10–16] financial problems [17] and employee performance [18]. Competent management deficiencies affect both the investor and the contractor [19,20].

Regarding railway projects as in construction, the identification of risk factors depends on the type of effects considered in the analysis (safety, costs, construction completion date delays) and the stage of the investment (design, construction) [2,4,21].

Attempts to eliminate potential risks are a key issue of construction projects at the risk management stage. In recent years, researchers and practitioners have made numerous efforts to count the assessment of the impact of potential risk factors when forecasting the implementation time of construction projects. Knowing the links between the risk factors, the strength of their interaction and their impact on the risk analysed is the basis for taking more targeted measures to decrease the level of risk. Multi-criteria analysis is an adequate method to evaluate factors and rank them by defining their weights. Most methods only specify weights on the basis of direct assessments by experts without examining the mutual influence between the factors [22]. A multi-criteria analysis method allows to consider the interaction of factors is the DEcision MAking Trial and Evaluation Laboratory (DEMATEL). This methods, based on the relationship between effective factors, allows to determine the strength of influence and the scale of dependence for each factor and to visualize this structure using an influence matrix or appropriate directed graphs. This method not only transforms the interdependence relationships into a cause-and-effect group through the matrix, but also finds the critical factors of the complex system through the influence relationship diagram. DEMATEL is used in solving problems in areas such as: business management, shaping products and services, transport, energy, medicine, finance, banking, education, information systems, environmental engineering and construction. [23,24]. In issues related to risk analysis in construction, this method was successfully applied in the work of Dytczak et al. [25] to identify the causes of building structure failure. It was also used as an effective tool of the risk analysis of tunnel engineering construction [26]. In the work of Mirosław-Swiatek et al. [27] it was used in the analysis of factors affecting the safety of flood embankments. The DEMATEL method was applied by Stoilova [28] to analyse the importance and the relations between the criteria and to establish the weights of the criteria for assesing the transport technology for passenger transport by railway and road. Farooq et al. [29] used DEMATEL to determine weights for smart urban mobility evaluation criteria in Beijing. Farooq’s research demonstrates the congruence between the evaluation of weights from DEMATEL and the Analityc Hierarchy Process (AHP) method, which is often used in multi-criteria analysis. There is research in which the two methods are combined by using DEMATEL to determine weights [30].

As mentioned above, railway investments, depending on the stage of implementation, are characterized by various risk factors. In this research we analyse the factors associated with the construction stage. While the risk factors themselves are widely discussed in the literature [3], there are no studies that analyse the relationship, importance, influence and impact of factors on each other.

The cause-and-effect model of factors influencing the risks associated with railway investments in Poland described in this publication, is based on factors identified in previous studies [22]. The risk factors were determined as a result of the study carried out with the participation of experts implementing one of the most complex railway investments in Poland. The DEMATEL method was applied to build this model, and the ranking of factors was performed on the basis of weights, and their estimation is an element of this method. The estimated weights for the risk factors were compared with the weights that were directly determined by the expert group and used in Kowalski’s research [3,22].

The novelty in this study is the assignment of weights (ranks) to the analysed risk factors and investigation of mutual influences of these factors. This research attempts to fill the gap in this area and can be used in other countries with similar risk factors for railway investments. This is extremely important in order to optimise the use of funds allocated for rail investment in Poland.

Due to the deficiencies in the literature of similar studies Thus, taking into account only factors typical of railway investments, the paper does not perform analyses indicating how the used in the study factors align or differ from those in other studies on railway investments in poland.

## Materials and methods

### Risk factors for railway investment (build stage)

The risk factors that pose the greatest threat to railway investment implementation for the build stage are listed in Table 1 and are consistent with the factors identified in the work of Kowalski [22]. Eleven key factors were identified by a group of experts with many years of successful experience in the implementation of railway investments, including scientists, contractors and investors.

**Table 1:** Risk factors for railway investment (build stage) Source: [22].

Risk factors	Description of a studied factors
**a1**	Errors in the preparation of tender documents (SIWZ*, OPZ*, PFU*).
**a2**	Improperly estimated time of completion of the investment by the Employer.
**a3**	Too many external institutions involved in the investment process.
**a4**	Terms of the contract not adapted to the contract specificity
**a5**	Investment costs incorrectly estimated by the Contractor
**a6**	Difficulties in the preparation, in terms of formal, legal and technical areas for investment.
**a7**	Internal regulations of PKP Polskie Koleje Państwowe S.A. (Polish State Railways) not coordinated with the provisions of contracts.
**a8**	Errors in project documentation
**a9**	Problems with the supply of materials and other resources
**a10**	Awarding shorter track closures to the contractor.
**a11**	Problems with outdated geodetic materials (numerous collisions with uninventoried underground infrastructure)

*SIWZ—contract terms and conditions; OPZ—description of the subject matter of the contract; PFU—functional-utility program.

**a1: Errors in the preparation of tender documents** result primarily from errors in the tender documents. In the Functional and Utility Program (FFU) itself, as well as in the Specification of Essential Terms of the Procurement (SIWZ), it is possible to identify probable causes of threats that may occur at the design and implementation stages. Examples include missing documents confirming the Employer’s legal title to use the land for construction purposes, outdated arrangements with external stakeholders or errors in the conceptual design.

**a2: Improperly estimated time of completion of the investment by the Employer** is a consequence of incorrect analyzes at the initiation stage of a given contract. Problems with incorrect estimation of implementation deadlines may be related to the lack of sufficient technical knowledge of the team preparing application documents for funding from the European Union.

**a3: Too many external institutions involved in the investment process** is a risk factor whose scale of impact cannot always be determined at the stage of the tender procedure. It happens that external institutions, whose participation is identified only after the design documentation has been developed, have unclear maximum deadlines within which they should make arrangements for the submitted design documentation. In Poland, an example may be companies belonging to the PKP Polskie Koleje Państwowe S.A. (Polish State Railways) capital group.

**a4: Terms of the contract not adapted to the contract specificity**. An example of failure to adapt contract provisions to the conditions prevailing in a given project is the need to construct engineering facilities located partly outside the Employer’s premises (railway area), where the manager of such area, who is not also a party to the contract, has a direct impact on the technical implementation and completion dates. Another major concern may be the incorrect selection of FIDIC (Federation Internationale des Ingenieurs-Conseils) Contract Conditions for a given investment. Not in all cases, implementing investments in accordance with the FIDIC Yellow Book procedures is the right solution.

**a5: Investment costs incorrectly estimated by the Contractor** result primarily from inappropriate internal procedures of the Contractor, regulating the process of preparing the investment for implementation.

**a6: Difficulties in the preparation, in terms of formal, legal and technical areas for investment** are mainly related to formal errors occurring during the preparation of the tender procedure. An example may be negligence towards further stakeholders, where the temporary occupation of their areas is necessary for the implementation of works.

**a7: Internal regulations of PKP Polskie Koleje Państowe S.A. (Polish State Railways) not coordinated with the provisions of contracts**. They are related to the role and possibility of significant influence on the process of agreeing project documentation of the Ordering Party’s internal organizational unit, i.e. the Investment Project Assessment Team (ZOPI). According to general knowledge, ZOPI has the obligation and authority to agree and adopt solutions for design studies regarding railway investments. On the other hand, it is not one of the parties to the contract, especially one implemented on the basis of the FIDIC Code. Moreover, arrangements with ZOPI, which consist of members without appropriate construction licenses and who are not directly responsible for and related to the implementation of the contract, may result in the need for constant detailing of the conceptual design and endless explanations, which should be resolved at the stage of the detailed design.

**a8: Errors in project documentation** may in particular refer to errors in the conceptual design documentation from the tender stage, which should be the starting material for the development of architectural and construction documentation by the designer. Unfortunately, the solutions indicated in the documentation in question (conceptual) often become outdated after signing the contract.

**a9: Problems with the supply of materials and other resources** may result from situations beyond the control of the parties to the contract, e.g. the War in Ukraine and/or from the Contractor’s organizational errors.

**a10: Awarding shorter track closures to the contractor.** Most railway investments are carried out under pain of maintaining train traffic at all rebuilt railway stations. In order to carry out the works, the Contractor to submit a request to the relevant units of PKP Polskie Koleje Państwowe S.A. (Polish State Railways) for temporary track closures. These closures enable performance of works that must be coordinated across industries. Unfortunately, approvals for track closures are largely withdrawn, sometimes several hours before the start of works.

**a11: Problems with outdated geodetic materials (numerous collisions with uninventoried underground infrastructure)** result primarily from the lack of documents in the PKP Polskie Koleje Państwowe S.A. (Polish State Railways) archives confirming the inventory of underground infrastructure from the 19^th^ and 20^th^ centuries.

### DEMATEL method

DEMATEL is a comprehensive a multi-criteria analysis method that allows to analyze complex dependencies in the system. This method transforms the interrelationships between factors into an understandable structural model of the system and divides them into a group of causes and a group of effects [31]. The developed cause and effect model can be presented in the form of a diagram. It allows for the description of the interrelationships between factors, determining the key factors considering the causal relationships and interrelationship degree between the factors and calculating the weights of factors by considering the interrelationships and factors impact levels. The basis of the DEMATEL method is the comparison of events (in this case the risk factors listed in Table 1) in pairs in terms of causality (direct impact). A rating scale of 0–3 is most often used to express the intensity of direct influence relationships. Its individual levels correspond to a gradual increase in the intensity of the influence relationship, where: 0 –no influence of the first event on the second event; 1 –low influence of the first event on the second event; 2 –significant influence of the first event on the second event; 3 –very high influence of the first event on the second event. The set of assessments of direct impact relationships allows, in mathematical transformations, the construction of a direct influence matrix *A*. From this, a matrix *T* of the total (both direct and indirect) influence is calculated. Its elements allow the values of two characteristic indicators to be determined: *Prominace* and *Relations*, identifying the nature of the factors under consideration in terms of their role in the process of determining the structure of influence of the factors (causes group) and their influence on other factors (effects group), respectively. A visual demonstration of cause-and-effect model is the Impact-Relations Map (IRM). As a result of the *T* matrix transformations, a net influence matrix—*NeT*—is developed, the graphical representation of which is the map of the total net influence, showing the strength of influence of one factor on another factor. Building the classic DEMATEL model can be formulated as follows [23,28].

Step 1—Generate the direct-influence matrix *A*.
  In this step, a direct influence matrix *A* = [*a*_*ij*_]_*n*×*n*_ is generated. Elements of matrix *A* indicate the direct influence that factor *a* has on factor *a*_*j*_ (Table 1), using an integer scale (0–3). Its individual rows are dedicated to the events that appear first in the comparison, and the columns to the events that appear second in the comparison. When determining matrix *A*, it is assumed that each factor can directly influence the other elements, but cannot influence itself, which means that all principal diagonal elements of matrix *A* are equal to zero. The values of the elements of the direct-influence matrix can be agreed jointly by experts or calculated as an average value from their opinions.
Step 2—Establish the normalized direct-influence matrix *X*.
  The normalized direct-influence matrix *X* = [*x*_*ij*_]_*n*×*n*_ is calculated by:
$$X=\frac{A}{s}$$ (1)
$$s=max\left({\text{max}}_{1\leq i\leq n}\sum_{j=1}^{n}a_{ij,}\underset{1\leq j\leq n}{\text{max}}\;\sum_{i=1}^{n}a_{ij,}\right)$$ (2)
Step 3—Construct the total–influence matrix *T*.
  Then (on the basis of matrix *X*), the total-influence matrix *T* = [*t*_*ij*_]_*n*×*n*_ is determined. It is calculated by summing the direct effects and all of the indirect effects by:
$$T=X{\left(I-X\right)}^{-1}$$ (3)
  where: *I*–identity matrix;
  The total influence matrix *T* contains information about the intensity of the total influence between factors.
Step 4—Calculation of the direct and indirect effects between factors.
  Two vectors *R* and *C*, representing the sum of the rows and the sum of the columns from the total-influence matrix *T*, are defined by the following terms:
$$Ri={\left[r_{i}\right]}_{n\times 1}={\left[\sum_{j=1}^{n}t_{ij}\right]}_{n\times 1}$$ (4)
$$Ci={\left[c_{j}\right]}_{1\times n}={\left[\sum_{i=1}^{n}t_{ij}\right]}_{1\times n}$$ (5)
  The *R*_*i*_ (influential power) index is the sum of the *i*-th row of the *T* matrix and describes the sum of direct and indirect influences (effects) exerted by factor *i* on other factors. Similarly, *C*_*i*_ (dependency) is the sum of the *i*-th column in the *T* matrix and represents the sum of the direct and indirect effects of the *T* matrix that factor *a*_*i*_ receives from other factors. It is therefore the value of the total, direct and indirect effects that the factors have on the analyzed factor.
  Based on the values of *R*_*i*_ and *C*_*i*_, a *Prominence* (*R*_*i*_ *+ C*_*i*_) and *Relation* (*R*_*i*_*—C*_*i*_) index is determined for each risk factor *a*_*i*_. *Prominence* describes the strength of influence given and received by a given factor and describes its role in determining the total influence of factors. Relation shows the net effect that a given factor contributes to the system. If *Relation* is positive then the factor *a*_*i*_ belongs to the causal group (has an impact on the system). If *Relation* is negative then the factor *a*_*i*_ is the net effect of the other elements of the system on it and is included in the effect group.
$$Prominence=R_{i}+C_{i}$$ (6)
$$Relation^{-}=R_{i}-C_{i}$$ (7)
Step 5—Determination of normalised degree of influence of each factor.
  On the basis of the *Prominence* it is possible to determine the normalised degree of impact (weighs of factor) for each factor, according to formula 8.
$$w_{i}=\frac{R_{i}+C_{i}}{\sum_{i=1}^{n}\;R_{i}+C_{i}}$$ (8)
Step 6—Development of the influential relation map (IRM).
  The influential relation map (IRM) is created by mapping the set of two indexes (*R+C*, *R-C*; Eqs: 4, 5, 6 and 7), that are calculated from the *T* matrix. IRM enables the visualization of complex causal relationships among factors. The resulting structure of total influence is often very complex because it represents all the connections between the elements of the system. To simplify it, a reduction is used, which involves filtering out irrelevant connections by eliminating from the *T* matrix connections with values lower than the assumed positive threshold of the total influence *θ*. Only those elements from the *T* matrix whose values are greater than *θ* are selected to represent their correlations in the IRM diagram. In the literature, the threshold value *θ* is usually determined by experts’ methods [23] or the average of all elements in the matrix *T* [32].
  Usually, the factors in the complicated system are grouped, depending on the value of *R+C* and *R-C*, into four quadrants according to their locations in the IRM diagram [33–35]. Due to their location in a specific quadrant, factors are classified as: most important (*R*_*i*_-*C*_*i*_ is positive; *R*_*i*_*+C*_*i*_ is large), important (*R*_*i*_*-C*_*i*_ is positive; *R*_*i*_*+C*_*i*_ is small), independent (*R*_*i*_*-C*_*i*_ is negative; *R*_*i*_*+C*_*i*_ is small), indirect (*R*_*i*_*-C*_*i*_ is negative; *R*_*i*_*+C*_*i*_ is large).
Step 7—Determination of the net influence matrix–*NeT*. The net influence matrix–*NeT* = [*Net*_*ij*_]_*n*×*n*_ [36,37] is used to evaluate the strength of influence of one factor on another factor. *NeT* elements describe the net influence value of a given factor. The *NeT* matrix is calculated from the total influence matrix *T*, according to the following formula:
$$Net_{ij}=\begin{array}{c} t_{ij}-\;t_{ji}\;\;\;\;\;t_{ij}-\;t_{ji}>0\\ 0\;\;\;\;\;\;\;\;\;\;\;\;\;\;\;\;\;t_{ij}-\;t_{ji}\leq 0 \end{array}$$ (9)
  A positive value of *Net*_*ij*_ element corresponds to the role of *i*-th evenet as a cause and a negative one as an effect of the occurrence of the *j*-th event [25].
Step 8—Drawing the map of the total net influence
  Based on the total net influence matrix, an acyclic and asymmetric directed graph can be developed, called the total net influence map, showing the impact relationships between individual factors. The arcs of the graph indicate the factor under the influence of the other factor, and the type of line represents the intensity of the total influence, estimated from the values of the elements of the *NeT* matrix.

### Expert grup

The selection of experts resulted from their competences and experience in the field of risk analyzes occurring in railway investments. These were people implementing and supervising railway investments in Poland, and scientists involved in research and teaching in the field of engineering construction projects. Nine experts took part in the evaluation of pairwise comparisons in terms of influence between the factors. Among them four experts are from academia. The condition for the selection of the remaining experts was appropriate seniority (at a minimum 10 years) and a broad view of railroad investments, taking into account the risk factors involved and their consequences. In the end, fife experts were selected who, in addition to their technical knowledge and experience, have general administrative and economic experience. All respondents were Polish citizens. At the time of the survey, the surveyed experts were directly involved in the implementation of one of the most difficult railroad investments in the Mazowieckie Voivodeship, which ensured a high level of quality of the data obtained.

In the presented study, each of the nine experts conducted pairwise comparisons in terms of influence between factors (according to a 0–3 scale) and independently presented an individual direct influence matrix. The elements of direct influence matrix *A* were determined as an average value based on the experts’ opinions.

## Results

In order to identify the relationships between the studied risk factors using the DEMATEL method, the computational model described above was used. Risk factors were identified as presented in Table 1. The direct infuence matrix *A* was determined using the expert method. In the presented study, each of the nine experts conducted pairwise comparisons in terms of influence between factors (according to a 0–3 scale) and independently presented an individual direct influence matrix. The final form of matrix *A* was estimated by taking the arithmetic mean of the values given by each expert. As a result of calculations using the DEMATEL method, direct influence matrix *A* (Table 2), total influence matrix *T* (Table 3) were obtained.

**Table 2:** Direct influence matrix *A*.

**Risk factors**	**a1**	**a2**	**a3**	**a4**	**a5**	**a6**	**a7**	**a8**	**a9**	**a10**	**a11**
**a1**	0.000	2.222	1.556	2.556	2.222	2.222	1.667	2.333	2.222	1.889	1.889
**a2**	2.000	0.000	2.000	1.667	2.889	2.000	1.667	1.556	2.111	1.889	1.778
**a3**	2.556	2.333	0.000	1.778	1.778	3.000	2.222	2.667	0.667	1.444	2.333
**a4**	2.222	2.333	1.556	0.000	2.333	1.667	2.333	1.556	1.889	1.333	0.889
**a5**	1.222	1.556	0.444	1.444	0.000	0.778	1.222	1.222	2.222	1.111	1.111
**a6**	2.778	1.444	2.333	2.111	2.222	0.333	1.333	1.778	1.222	1.222	2.111
**a7**	2.222	2.111	1.889	2.667	2.556	2.111	0.000	2.667	1.000	2.556	1.444
**a8**	2.667	1.778	1.444	2.222	3.000	2.111	2.111	0.000	2.444	1.222	2.444
**a9**	1.333	1.778	1.000	1.000	2.000	1.111	1.000	1.111	0.000	1.222	0.667
**a10**	1.667	2.667	0.778	1.556	2.556	1.333	1.778	1.444	2.000	0.000	1.000
**a11**	2.667	2.111	1.667	1.000	2.111	2.111	1.444	3.000	1.111	0.889	0.000

**Table 3:** Total influence matrix *T* (threshold value *θ* = 0.295; bold type indicates all elements that are greater than *θ*).

**Risk factor**	**a1**	**a2**	**a3**	**a4**	**a5**	**a6**	**a7**	**a8**	**a9**	**a10**	**a11**	**Ri**
**a1**	**0,299**	**0,372**	0,274	**0,358**	**0,421**	**0,346**	**0,306**	**0,361**	**0,338**	0,288	**0,300**	3,663
**a2**	**0,357**	0,268	0,275	**0,309**	**0,422**	**0,321**	0,290	**0,316**	**0,316**	0,275	0,282	3,430
**a3**	**0,412**	**0,386**	0,223	**0,342**	**0,417**	**0,388**	**0,336**	**0,387**	0,288	0,281	**0,330**	3,791
**a4**	**0,349**	**0,343**	0,249	0,231	**0,386**	0,295	**0,302**	**0,301**	**0,296**	0,246	0,237	3,236
**a5**	0,227	0,234	0,146	0,213	0,202	0,187	0,193	0,211	0,239	0,175	0,179	2,204
**a6**	**0,381**	**0,320**	0,285	**0,321**	**0,391**	0,240	0,274	**0,321**	0,278	0,245	0,292	3,349
**a7**	**0,396**	**0,379**	0,293	**0,373**	**0,445**	**0,351**	0,251	**0,383**	**0,302**	**0,321**	0,293	3,787
**a8**	**0,408**	**0,362**	0,274	**0,352**	**0,455**	**0,347**	**0,327**	0,279	**0,351**	0,269	**0,326**	3,749
**a9**	0,231	0,242	0,167	0,197	0,279	0,200	0,185	0,207	0,152	0,179	0,164	2,203
**a10**	**0,304**	**0,333**	0,202	0,271	**0,369**	0,261	0,263	0,274	0,283	0,175	0,222	2,956
**a11**	**0,374**	**0,339**	0,259	0,278	**0,385**	**0,319**	0,275	**0,362**	0,273	0,231	0,209	3,304
**Ci**	3,740	3,576	2,647	3,245	4,173	3,255	3,001	3,400	3,115	2,684	2,834	***0*,*295***

The threshold of the total influence *θ* was calculated as the average of all elements in the matrix *T*.

Based on the *T* matrix, the influential power (*R*_*i*_), dependency (*C*_*i*_), Promineance (*R*_*i*_*+C*_*i*_), Relation (*R*_*i*_*-C*_*i*_) and weighs of factor (*w*_*i*_) indicators were calculated (Eqs 4–8), which are summarized in Table 4.

**Table 4:** Total effects and net effects, weighs and ranks for factors.

Factor	R	C	R + C	R—C	Group	w [%]	Rank
a1	3,663	3,740	7,403	-0,078	Affected	10,38	a1
a2	3,430	3,576	7,006	-0,147	Affected	9,82	a8
a3	3,791	2,647	6,438	1,144	Cause	9,02	a2
a4	3,236	3,245	6,481	-0,009	Affected	9,08	a7
a5	2,204	4,173	6,377	-1,969	Affected	8,94	a6
a6	3,349	3,255	6,604	0,094	Cause	9,26	a4
a7	3,787	3,001	6,788	0,786	Cause	9,51	a3
a8	3,749	3,400	7,149	0,349	Cause	10,02	a5
a9	2,203	3,115	5,318	-0,913	Affected	7,45	a11
a10	2,956	2,684	5,640	0,273	Cause	7,91	a10
a11	3,304	2,834	6,137	0,470	Cause	8,60	a9

(*R*_*mean*_ = 3.243 *C*_*mean*_ = 3.243; *(R+C)*_*mean*_ = 6.486; grey colour indicates values above the mean value).

The factor with the greatest direct and indirect impact (*R* index) on other factors is a3 –Too many external institutions involved in the investment process. The smallest effect on other factors is a5 –Investment costs incoretly estimated by the Contractor and a9 –Problems with the supply of materials and other resources. Values above the average for the total impact indicator (*R* index) are: a7 –Internal regulations of PKP Polskie Koleje Państwowe S.A. (Polish State Railways) not coordinated with the provisions of contracts, a8 –Errors in project documentation, a1 –Errors in the preparation of tender documents (SIWZ, OPZ, PFU), a2—Improperly estimated time of completion of the investment by the Employer, a6—Difficulties in the preparation, in terms of formal, legal and technical areas for investment, a11—Problems with outdated geodetic materials (numerous collisions with uninventoried underground infrastructure). The factor most dependent on the others (index *C*) is a5 –Investment costs incorretly estimated by the Contractor, and the most independent is a3 –Too many external institutions involved in the investment process. Values above the average are: a1 –Errors in the preparation of tender documents (SIWZ, OPZ, PFU), a8 –Errors in project documentation, a2 –Improperly estimated time of completion of the investment by the Employer, a7—Internal regulations of PKP Polskie Koleje Państwowe S.A. (Polish State Railways) not coordinated with the provisions of contracts, a6 –Difficulties in the preparation, in terms of formal, legal and technical areas for investment.

**Fig 1:**
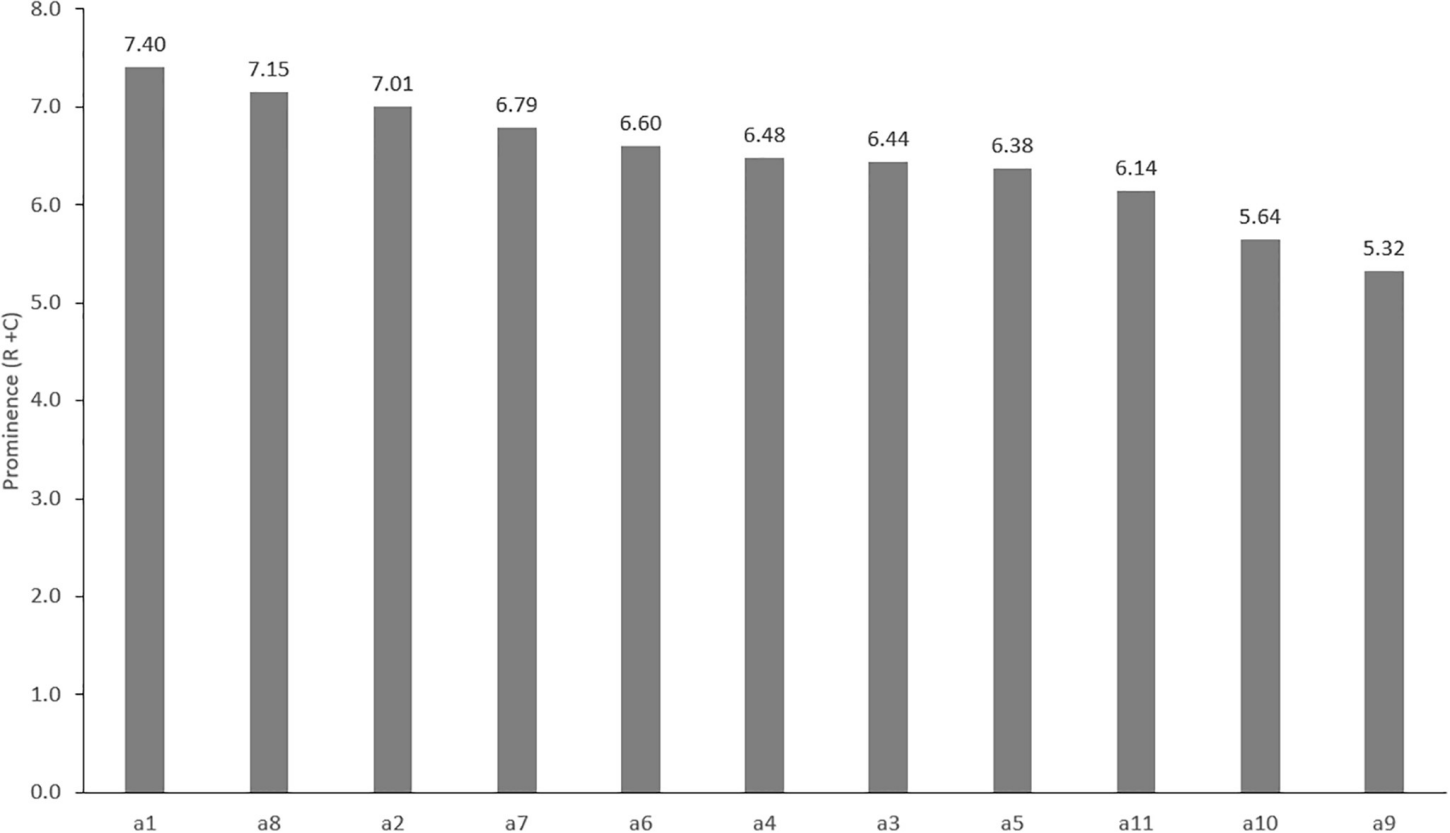
Prominence graph (R+C).

The analysis of Table 4 and Fig 1 shows that the highest value of *Prominence* is achieved by factor a1 ‒ Errors in the preparation of tender documents (SIWZ, OPZ, PFU), which proves its central role in the process of determining the total impact of the factors. Factors a8 ‒ Errors in project documentation and a2 ‒ Improperly estimated time of completion of the investment by the Employer are also highly influential. The following factors also achieve values above the average: a7 ‒ Internal regulations of PKP Polskie Koleje Państwowe S.A. (Polish State Railways) not coordinated with the provisions of contracts, a6 ‒ Difficulties in the preparation, in terms of formal, legal and technical areas for investment. The factors with the lowest impact are: a10 ‒ Awarding shorter track closures to the contractor, a9 ‒ Problems with the supply of materials and other resources.

**Fig 2:**
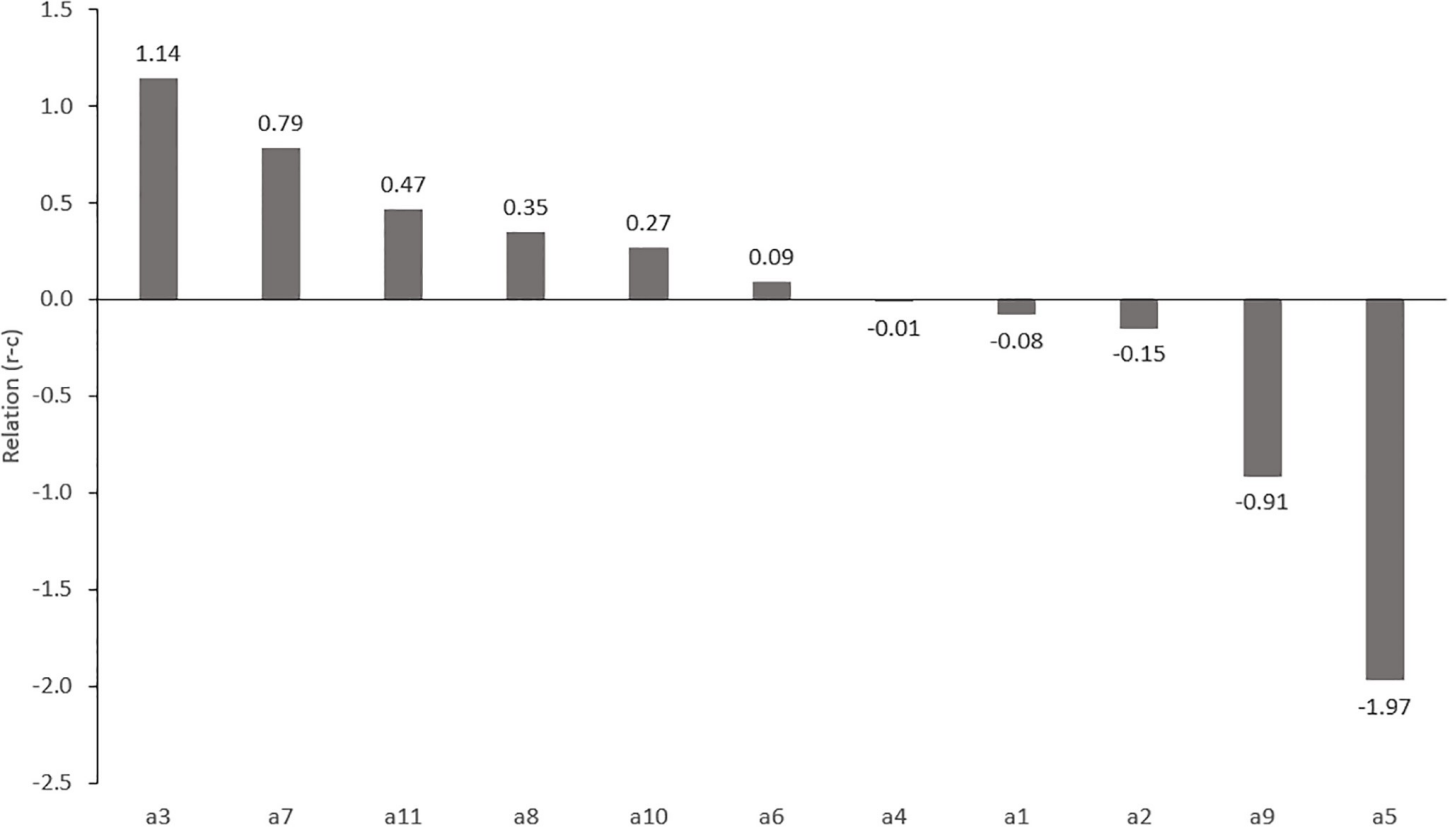
Relation graph (R-C).

The importance of the factors in terms of the *Relation* index is shown in Fig 2. The group of causes (*R*_*i*_*-C*_*i*_ > 0) includes the following five factors: a3, a7, a11, a8, a10, a6. By far the highest *Relation* value is a3, which proves that it has the greatest influence on other factors. Next comes the a7. The remaining 4 factors have a much smaller impact on the other factors. Factors a4, a9, a1, a2, a5 belong to the group of effects (*R*_*i*_*-C*_*i*_ < 0) and are influenced by causal factors. In this group, the factor with the greatest influence is a5. Factor a4 is the nearest to the center, it means that it is least influenced by the identified causal factors.

**Fig 3:**
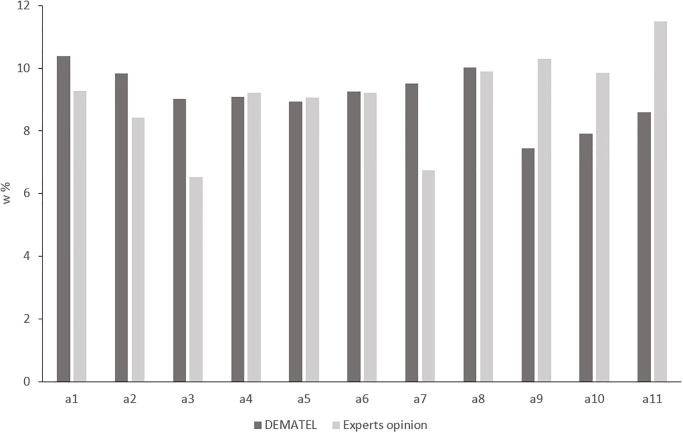
Normalised degree of factor importance.

The weights (Eq 8) calculated in the DEMATEL method for the individual risk factors are summarised in Table 4. They range from 7.45% to 10.38%. The factors of great importance are a1 (10.38%) and a8 (10.02%). The factors with the lowest impact are a9 (7.45%) and a10 (7.91%). Calculated with DEMATEL weights were compared (Fig 3) with the weights that, for the eleven risk factors analysed, were directly determined by the experts [22]. In Kowalski’s research [22], the experts assigned importance on a scale from 1 to 10, and the calculated weights range from 6.75% to 11.49%. The lowest percentage difference in weight values is 0.50% and is reached for a6. The greatest difference of 38.19% is found for a9. For four factors (a4, a5, a6, a8), the differences do not exceed 1.44%. In the 10–30% range, percentage differences occur for factors a1, a2, a10, a3 and a7. Only for factors a11 and a9 are the percentage differences greater than 30%.

**Fig 4:**
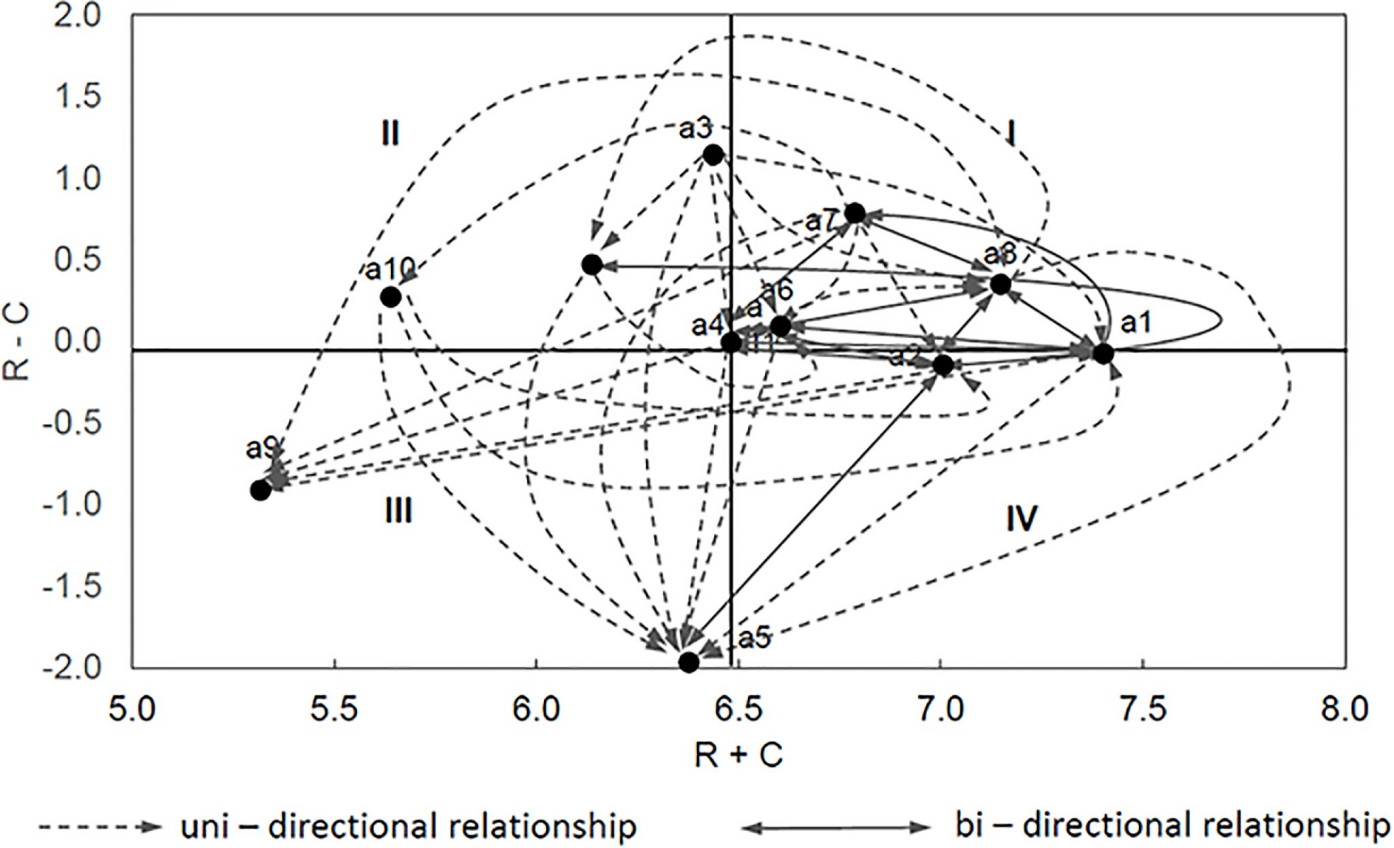
Influential relation map (IRM) of the factors (mean of R+C = 6.486).

Fig 4 shows the IRM diagram, which was developed on the basis of the *Prominence* (*R+C*) and Relation indicators (*R-C*) (Table 4). Factors a7, a8, a6 belong to group I (the most important factors) because they influence (*R*_*i*_*-C*_*i*_ *> 0*) the remaining factors (they are the causes) and at the same time significantly related to them *R*_*i*_*+ C*_*i*_ >(*R+C*)_*mean*_). Factors a3, a11, a10 belong to group II (important factors), they significantly influence the other factors (*R*_*i*_*-C*_*i*_ > 0), but weakly related to them (*R*_*i*_ *+ C*_*i*_ < (*R+C*)_*mean*_), so they can be treated in the system as autonomous causes. Group III (independent factors) includes three factors a9, a5 and a4. They are effects (*R*_*i*_*-C*_*i*_ < 0) and at the same time weakly related to other factors (*R*_*i*_*+C*_*i*_ < *(R+C)*_*mean*_). The remaining three factors a1, a2 belong to group IV (Indirectly factors). They are effects (*R*_*i*_*-C*_*i*_ < 0) and, at the same time, strongly related (*R*_*i*_*+C*_*i*_ >(*R+C*)_*mean*_) to other factors affecting the risk of failure of railway investments. IRM diagram maps the correlations between the risk factors by using solid lines and broken lines for two-way and one way significant relationships, respectivley. Only those relationships for which the elements of the *T* matrix (Table 3) are greater than the threshold value (*θ = 0*.*295*) are considered. Significant bi-directional relationship with the other factors occur for a7, a8 and a6. These factors are characterised by *Prominence* values above average and belong to the group of causes.

Table 5 shows the net influence between the studied risk factors. This relationship is used to assess the strength of the influence of one factor on another (Eq 9).

**Table 5:** Net influence matrix *NeT*.

**Risk factor**	**a1**	**a2**	**a3**	**a4**	**a5**	**a6**	**a7**	**a8**	**a9**	**a10**	**a11**
**a1**		0,015		0,009	0,194				0,106		
**a2**					0,189	0,001			0,075		
**a3**	0,139	0,111		0,093	0,271	0,103	0,043	0,113	0,121	0,078	0,071
**a4**		0,034			0,173				0,099		
**a5**											
**a6**	0,036			0,025	0,204				0,078		
**a7**	0,090	0,089		0,071	0,252	0,077		0,056	0,118	0,059	0,018
**a8**	0,047	0,046		0,051	0,245	0,026			0,144		
**a9**					0,041						
**a10**	0,016	0,058		0,026	0,195	0,015		0,005	0,104		
**a11**	0,074	0,057		0,041	0,206	0,028		0,036	0,109	0,009	

**Fig 5:**
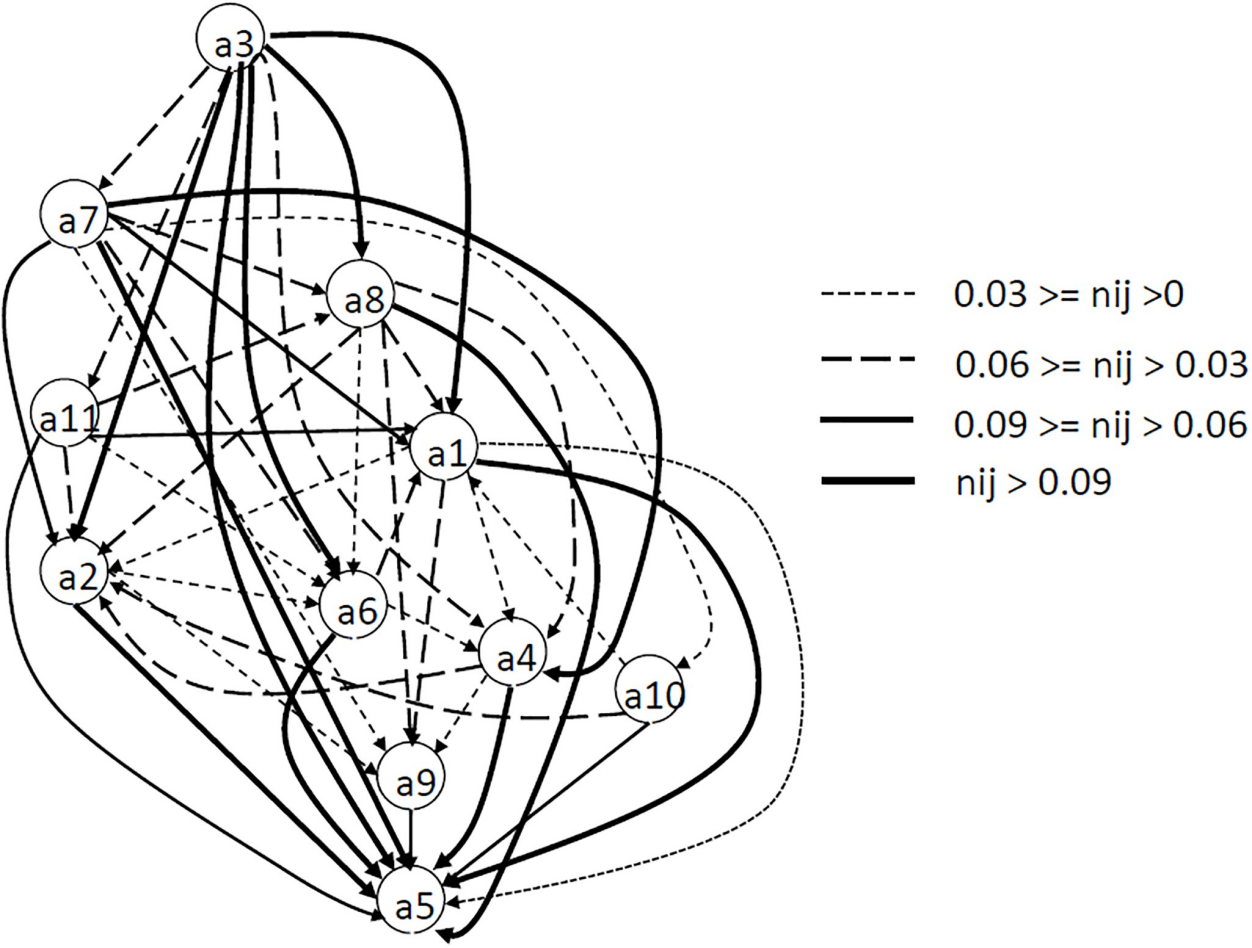
Map of the total net influence. Graph arcs indicate a factor under the influence of another factor and the type of line -total influence intensity—the value range for element *n*_*ij*_ in the NeT matrix.

The analysis of the values listed in the table above shows that, for example, factors a3 and a7 influence remaining factors to a greatest extent. Factors a5 and a9 practically does not influence any other factor, it is practically always the effect of other factors (Fig 4). Based on the *NeT* matrix, the total net influence map was developed (Fig 5). The strength of the influence of one factor on another is shown in four classes (0 < *Net*_*ij*_ ≤ 0.03 weak influence; 0.03 < *NeT*_*ij*_ ≤ 0.06 average influence; 0.06 < *NeT*_*ij*_ ≤ 0.09 strong influence; *NeT*_*ij*_ > 0.09 very strong influence). The developed map of the total influence of factors confirms the strongly causal nature of factors a3 and a7, which are located in the upper part of the map, and strongly consequential nature of factor a5, which is located at the bottom. Factor a3 has a very strong net impact on related factors. Five of the eight impacts exceed the lower limit (0.09) of the strongest net influence. The second factor a7 only exceeds 0.09 for two of the links *NetT*_*75*_ and *NetT*_*79*_. All eleven factors intensively influence a5, eight of them feature a very strong net influence.

An analysis of Figs 3 and 4 shows that the ranking of factors based on the value of the calculated weights is as follows: a1>a8>a2>a7>a6>a4>a3>a5>a11>a10>a9. According to Eq 8, the weights are estimated based on the value of *Prominence*. It shows the degree of being influenced by other factors and influences them as well. Fig 4 presents the cause and effect diagram and IRM for the factors. It shows that a8 is a key factor for risk problems. Factors a7, a6 have also high importance. All 3 factors are characterized by high *Relation* and *Prominence*. They are related to each other and to the other factors. This is particularly evident in Fig 5, where the significant influence of factor a7 on the others is shown. Among the cause group factors that are autonomous in the system (factors: a3, a11, a10) (Fig 4), factor a3 has the highest *Relation*. Its autonomy and strong influence on other factors is evidenced by Fig 5. In the group of independent factors (factors: a4, a5, a9) (Fig 4). factor a5 has the lowest *Relation* value. Its character, as an autonomous receiver, can be seen in Fig 5. It is very strongly influenced by all other factors. The Fig 4 demonstrates that factors a1, a2 have an indirect impact on the system. They are interpreted as intertwined receivers (low *Relation* but high *Prominence*) cannot be enhanced directly and must be influenced by other ones. This is confirmed by Fig 5, where they are located in the middle of the diagram and more factors have a net influence on them than they do on the others.

## Discussion

The basis of the DEMATEL method, like other methods used in multi-criteria analysis, is the subjective assessment of experts. In most applications of this method in its classic approach (Fontela et al., 1972), experts evaluate the direct influence between any two criteria by using a five-point scale (0—no influence; 1—low influence; 2—medium influence; 3—high influence; 4—very high influence). In our study, experts used a scale of 0–3, in which intermediate levels 1 and 2 describe a gradual transition (low influence, significant influence) from no influence to very high influence of one factor on another. According to experts, the use of a 0–3 scale significantly simplifies a clear pairwise comparison of the analyzed 11 risk factors. A four-point scale for comparisons between criteria is often used in various studies that use the DEMATEL method [24,25,27].

The assessment of the importance of risk factors on railway investment implementation for the build stage was made on the basis of factor weights, calculated using the classical DEMATEL technique, in which the weights are determined based on normalized prominence (*R+C*) (Eq 8). Despite various modifications introduced by some authors in calculating criteria weights in DEMATEL [24,38,39], the classic method of estimating them is widely used [28,29]. The calculated criteria weights are characterized by little variability. Their average value is 9.09% (Fig 3) and the standard deviation is 0.87%. This distribution of weights, which vary in the range from 7.45% to 10.38%, indicates that the factors are characterized by similar importance weights for factors.

Due to the lack of research in the literature related to the construction of a cause-and-effect model of risk factors for railway investment implementation, it is difficult to compare our results against the background of other studies. The results of previous research conducted by Kowalski [22] were used to validate the importance calculated by the DEMATEL (Eq 8) for individual risk factors. The weights determined in accordance with Eq 8 were compared with the weights calculated on the basis of direct opinions of experts. The weights assigned by experts are one of the core issues of the Railway Matrix of Risk Factors (RMRF) method, developed by Kowalski to analyze risk for railway investment [3,22]. In Kowalski’s research, experts directly answered on a scale from 1 to 10 the question of what influence a specific factor has on the investment time and cost. Questionairre involved large groups of 85 experts with extensive professional experience. The average percentage difference between the scales is 16.60% and proves that the results of both methods are close (Fig 3). In the DEMATEL method, factors a1, a2, a3, and a7 were more important than in the opinion of experts. The opposite situation occurs in the case of factors a9, a10, a11. The remaining factors (a4, a5, a6) have similar weights in both methods. In the DEMATEL method, the weights change to a lesser extent (7.45%–10.38%) than the weights determined directly by experts (6.75% -11.49%). Experts significantly overestimate the role of the a11 factor, which they give the greatest importance in their assessment. Perhaps this is due to the fact that it is hard for the experts to detect indirect connections between the factors that the DEMATEL method determines analytically, based on the computational algorithm. In our opinion, experts also overestimate the importance of factors that have a high probability of occurring. According to Leśniak and Janowiec [4], the probability of a11 occurrence is 76%, and experts attach the greatest importance to this factor.

Taking into account the very wide scope of research conducted by Kowalski [22], in our analysis we used the 11 factors he identified that influence the risk for railway investment implementation for the build stage. The ranking of factors a1>a8>a2>a7>a6>a4>a3>a5>a11>a10>a9 obtained in the DEMATEL method differs from the ranking determined on the basis of direct expert opinions in the RMRF method (a11>a9>a8>a10>a1>a6>a4>a5>a2>a7>). However, most importantly, the weights assigned to the factors are close in both methods. Despite the fact that there are different groups of experts in both methods (both in terms of their composition and number), the similar weight values (DEMATEL vs. RMRF) prove the great application potential of DEMATEL, in which a much simpler task was set for a smaller number of experts, based on direct pairwise comparison of factors. This is one advantage of the DEMATAL method. It can be combined subjectively and objectively to make a comprehensive assessment. Another advantage of the DEMATEL method is the fact that experts estimate the direct impact for each pair of factors only once, and any indirect connections are the result of computational analyzes used in this method. This approach eliminates potential errors resulting from engaging experts on a broader scale. In our future studies, we will combine both methods by using DEMATEL to determine the weights for risk factors in the RMRF method. This will reduce the impact of experts’ subjectivity in assessing the importance of factors on risk. Combining DEMATEL with other multi-criteria analysis methods to determine weights for criteria is often used in the literature [29,40].

The obtained cause-and-effect model of connections between factors presented using IRM and Map of the total net influence (Figs 4 and 5) indicates the dominant nature of factors a7—Internal regulations of PKP Polskie Koleje Państwowe S.A. (Polish State Railways) not coordinated with the provisions of contracts and a8 ‒ Errors in project documentation. They are the causes strongly related to many factors and have a large net influence on other factors. Errors in design documentation are among the most common events. Leśniak and Janowiec [4] state that the probability of their occurrence is 97%. The Map of the total net influence shows that factor a3—Too many external institutions involved in the investment process (located in the upper part of the diagram), has the greatest net influence on other factors, and its location in IRM (*R+C* = 6.481 close to the average value of 6.486) indicates that it belongs to the transitional group between important factors and the most important factors in the structure of connections. Factors a2, a1, a4, a6 have a double role (partly effect—partly cause) and their *R-C* value is close to zero and varies in the range from -0.15 to 0.09 (Fig 2). This is particularly visible in the Map of the total net influence, where these factors are located in the middle part of the diagram. Factors a10 and a11 are causes (Fig 4) that have a small net effect on the associated factors (Fig 5). The definite effects are factors a5 and a9 (Fig 4). Due to the value of *R+C* = 6.377 located close to the average value of 6.486 (Fig 4), factor a5 is related to many factors that have a very high influence on it. It can also be defined as a dual factor, which is partly independent and partly an indirect factor. The conducted research fills the knowledge gap in terms of quantitative description of the importance of risk factors for railway investments in Poland and investigation of factors mutual influences. This is a very important issue and in the last few years, numerous legal regulations have been introduced (European standards, national instructions) aimed at unifying the requirements for research and design calculations, as well as the scope and format of design documentation (factor a7). Such standardization is also intended to limit (to the most professional and competent) the number of institutions involved in the investment process (factor a3). These activities can speed up the investment process, and shortening it may be crucial in determining the most likely investment cost (factor a5). Eliminating problems with the supply of materials is possible by implementing alternative design solutions that provide for the use of replacement building materials that meet the technical requirements (a5). This is of great importance for both accelerating and eliminating delays in ongoing railway investments. Identifying factors a3, a7 and errors in project documentation (factor a8), as the most important factors is crucial for practice. Previously mentioned activities like standardizing research and design (introduced regulations) unifying the requirements for the design documentation can also improve the quality of the documentation by minimizing or eliminating errors in project documentation (factor a8).

The weights of the individual factors, determined by the DEMATEL method, are the result of mathematical transformations of the direct influence matrix, which is derived on the assumption that the relative weights of experts are equally important. In practice, each expert has unique characteristics in terms of knowledge, skills, experience and personality, which means that different weights of experts should be assigned to their influence on the final results. In the interrelationship evaluation process, some experts may assign unduly high or unduly low values depending on their preferences. Therefore, advanced DEMATEL methods should be developed in the future to mitigate the impact of unfair arguments on the decision results. It is crucial to propose more objective and efficient methods to establish key parameters in DEMATEL, such as the threshold value considered in the causality diagram in IRM and to correctly account for the influence of both the prominence and relation index in the method of calculating weights.

The proposed DEMATEL model for determining weights for risk factors may be used in the future in the Railway Matrix of Risk Factors method, intended for estimating risk for railway investment. The identified structure of connections between factors can be the basis for the development of applications of risk models for railway investments, the basis of which is the identification of cause-effect connections (for example, models based on Bayesian Belief Network [4]).

Regardless of the method used, the analysis of 11 factors by experts seems to be quite a complex issue, so it may be worth using the method of grouping factors, which leads to reducing the size of the issue, and then conducting a DEMATEL analysis for the groups and within them. This type of approach was successfully used in other studies [28,41].

The results of the analysis indicate that recommendations or strategies should be developed in the future in order to minimise the negative effects of factors a8, a7, a6 or a3. This could be achieved through introducing additional studies and analyses at the pre-design stage, changing the form of documentation—introducing uniform documentation formats across various branches. Creation of databases that will enable the application of artificial intelligence for certain decisions.

## Conclusions

The analysis of cause and effect relationships of risk factors for failure of railway investments presented in the article, performed using the DEMATEL technique, allowed to classify them as cause or effect factors. The group of cause factors includes in decreasing order: errors in project documentation (a8), internal regulations of PKP Polskie Koleje Państwowe S.A. (Polish State Railways) not coordinated with the provisions of contracts (a7), difficulties in the preparation, in terms of formal, legal and technical areas for investment (a6), too many external institutions involved in the investment process (a3), problems with outdated geodetic materials (a11) and awarding shorter track closures to the contractor (a10).

Other factors ranked in decreasing order: errors in the preparation of tender documents (SIWZ, OPZ, PFU) (a1), improperly estimated time of completion of the investment by the Employer (a2), terms of the contract not adapted to the contract specificity (a4), investment costs incorrectly estimated by the Contractor (a5), problems with the supply of materials and other resources (a9) belong to groups of effects and are influenced by causal factors. Among this group of factors, investment costs incorrectly estimated by the Contractor (a5) is the least influencing factor among all identified factors.

Due to the value of the *Relation* indicator close to zero, factors a2, a1, a4, a6 play a double role (partly the effect—partly the cause).

The highest value of the prominence index (causes group) for the errors in project documentation factor (a8) indicates that it is the most important for the implementation of railway investments. Important factors are: problems with outdated geodetic materials (a11) and awarding shorter track closures to the contractor (a10).

Problems with the supply of materials and other resources (a9) belongs and to the group of independent factors that have little impact on the others factors.

Factors strongly related to the remaining factors but being an effect of the influence of the remaining factors are: errors in the preparation of tender documents (SIWZ, OPZ, PFU) (a1), investment costs incorrectly estimated by the Contractor (a5) and improperly estimated time of completion of the investment by the Employer (a2).

The application of the DEMATEL method made it possible not only to identify the relations between the factors, but also, by calculating the weights, to determine the importance of the factor. It was found that the factors errors in the preparation of tender documents (SIWZ, OPZ, PFU) (10.38%), errors in project documentation (10.02%), improperly estimated time of completion of the investment by the Employer (9.82%), internal regulations of PKP Polskie Koleje Państwowe S.A. (Polish State Railways) not coordinated with the provisions of contracts (9.51%) have the highest degree of importance. The weights calculated in DEMATEL were validated by the importance of factors, which in earlier studies were determined directly by experts. The weights assigned to the factors are close in both methods, the percentage difference not exceeding 16.60%.

The manuscript addresses the practical implications of the research for risk management in railway projects. It is therefore important to develop specific guidelines in the future for combating negative effects or preventing individual risk factors. The proposed DEMATEL model for determining weightts for risk factors may be used in the future in the Railway Matrix of Risk Factors method, intended for estimating the risk of railway investments. The identified structure of connections between factors may be the basis for the development of applications of railway investment risk models based on the identification of cause-effect connections (for example, models based on Bayesian Belief Network).
